# Disinfection By-products: A Question of Balance

**DOI:** 10.1289/ehp.1003053

**Published:** 2010-11

**Authors:** Rita Schoeny

**Affiliations:** Office of Water, U.S. Environmental Protection Agency, E-mail: schoeny.rita@epa.gov

By all accounts, disinfection of drinking water is one of the major public health triumphs of the 20th century. No human endeavor, however, is without risk; more than 30 years ago it was determined that chlorination of water resulted in formation of disinfection by-products (DBPs). Several of these, such as the trihalomethanes (THMs) and haloacetic acids (HAAs), were subsequently observed to produce cancer in animal models and to have other toxic end points, such as reproductive and developmental effects. Moreover, some of these end points have been associated with consumption of disinfected water in human populations; in particular, an elevated risk for bladder cancer has been observed in published epidemiology studies. Many governments have set limits on the amount of DBPs that may be present in drinking water produced by public systems. The U.S. Environmental Protection Agency (EPA), for example, has regulatory limits of 80 μg total THMs and 60 μg total HAA per liter of water (Richardson et al. 2007). However, regulations must also ensure that efforts to reduce DBPs do not result in water that is impaired due to microbial contamination: This is the microbe–DBP balancing act of minimizing risk while maximizing beneficial effects.

In this issue of *EHP*, a suite of four articles builds upon the hundreds of studies (see Richardson et al. 2007) reported since the first report of the genotoxicity of disinfected water (Loper et al 1978). These articles include a case–control study of gene–environment interaction in bladder cancer and three studies of DBP exposures in swimming pool water and biomarkers of their potential effects among swimmers. In the case–control study, Cantor et al. (2010) estimated associations between bladder cancer and individual DBP exposure estimates based on THM measurements in municipal water sources that were linked to residential history. The authors report that participants who carried a glutathione *S*-transferase zeta-1 (*GSTZ1*) rs1046428 polymorphism and were missing one or both copies of *GSTT1* appeared to be particularly susceptible to bladder cancer when exposed to relatively high levels of THMs in water [odds ratio = 5.9 (95% confidence interval, 1.8–19.0) for > 49 μg versus ≤ 8 μg THMs/L]. The two susceptibility alleles occurred together in 29% of the Spanish study population (Cantor et al. 2010).

Richardson et al. (2010) identified > 100 DBPs in water samples from two indoor pools in Barcelona, Spain (one chlorinated and one brominated). The samples included some nitrogen-containing DBPs that had not been found previously in drinking water, which the authors suggest may have formed due to the presence of nitrogen sources in pool water, such as urine or sweat. Nonetheless, they report that mutagenic potencies of the pool water (based on the *Salmonella* assay) were similar to that of typical drinking water.

Kogevinas et al. (2010) report that bathers in the chlorinated Barcelona pool had an increased frequency of micronuclei in peripheral blood cells (a marker of DNA damage) and evidence of increased systemic exposure to mutagens (based on urinary mutagenicity using the *Salmonella* assay) after a 40-min swim. Both end points were also associated with the concentrations of brominated THMs—but not chloroform—in the exhaled breath of the swimmers. In addition, Font-Ribera et al. (2010) report that Clara cell secretor protein (CC16), an indicator of increased lung epithelium permeability, was increased after swimming in the same chlorinated pool. As noted by the authors, alterations in Clara cell permeability are thought to play a role in the development of asthma, which has been linked to indoor swimming pool exposure. However, both exercise and DBP exposure could have contributed to the increase in CC16 after swimming.

Collectively, these studies provide the clearest evidence to date that disinfected water might be genotoxic and carcinogenic to humans, and that genotype may be a critical factor in susceptibility to bladder cancer in individuals exposed to DBPs. However, these researchers emphasize that their results need to be replicated and note that another case–control bladder cancer study is currently under way by the U.S. National Cancer Institute (National Institutes of Health).

How can these data be used by regulators? I suggest again that it is a matter of balance. Swimming is generally considered to be a health-enhancing activity; it can be a good aerobic exercise, contribute to flexibility and muscle strength, and can have positive social aspects. However, after water leaves a public water system and enters a pool, its quality is not regulated at the national level in the United States as it is in some countries, and the Safe Drinking Water Act makes no stipulation that tap water meet any additional requirements for use in pools or spas. Instead, pool water quality is managed at the most local level in the United States, usually by state or local public health departments, and sometimes by pool managers or lifeguards. In the pool balancing act, one must weigh immediate hazards that might result from exposure to microbial pathogens if adequate disinfection levels are not maintained against potential long-term hazards associated with exposure to DBPs. In addition, regulators must also consider the cost of delivering high-quality water to the consumer. One option to decrease the responsibility and expense incurred by public drinking water systems is to improve the quality of source water through pollution prevention and precursor removal. However, a source reduction approach is challenging for swimming pools, because the swimmers themselves are the largest source of nitrogenous substrates and a substantial source of organic matter necessary for DBP formation.

What is next for regulatory groups? It may be time to consider the feasibility of a population-based risk assessment for DBPs based on the evolving knowledge of genetic susceptibility to DBP-associated cancer. Any risk management approach for DBPs must be balanced against risk of waterborne disease, without exquisite information on genetic or life-stage susceptibility for these diseases. In addition, increased emphasis on DBPs, or any class of contaminants, will be balanced against the need for research and risk assessment for chemicals not currently addressed by regulations.

Tools are available to help in sorting priorities for risk assessment in support of risk management. Also in this issue of *EHP,* Claxton et al. (2010) review the 40-year history of the *Salmonella* mutagenicity assay and discuss that history informs the development of 21st century toxicology. The *Salmonella* mutagenicity assay was used > 30 years ago in the first report of the mutagenic potential of drinking water (Loper et al. 1978), and it continues to be used in hazard identification, as evidenced by the current swimmer studies. In this century, we need to use all of the tools available to us—advanced analytical methods, genomic end points, and engineering approaches—to guarantee the delivery to all of water that is safe for drinking, bathing, and swimming.

## References

[b1-ehp-118-a466] Cantor KP, Villanueva CM, Silverman DT, Figueroa JD, Real FX, Garcia-Closas M (2010). Polymorphisms in *GSTT1, GSTZ1*, and *CYP2E1*, disinfection by-products, and risk of bladder cancer in Spain. Environ Health Perspect.

[b2-ehp-118-a466] Claxton LD, Umbuzeiro GA, DeMarini DM (2010). The *Salmonella* mutagenicity assay: the stethoscope of genetic toxicology for the 21st century. Environ Health Perspect.

[b3-ehp-118-a466] Font-Ribera L, Kogevinas M, Zock J-P, Gómez FP, Barreiro E, Nieuwenhuijsen MJ (2010). Short-term changes in respiratory biomarkers after swimming in a chlorinated pool. Environ Health Perspect.

[b4-ehp-118-a466] Kogevinas M, Villanueva CM, Font-Ribera L, Liviac D, Bustamante M, Espinoza F (2010). Genotoxic effects in swimmers exposed to disinfection by-products in indoor swimming pools. Environ Health Perspect.

[b5-ehp-118-a466] Loper JC, Lang DR, Schoeny RS, Richmond BB, Gallagher PM, Smith CC (1978). Residue organic mixtures from drinking water show *in vitro* mutagenic and transforming activity. J Toxicol Environ Health.

[b6-ehp-118-a466] Richardson SD, DeMarini DM, Kogevinas M, Fernandez P, Marco E, Lourencetti C (2010). What’s in the pool? A comprehensive identification of disinfection by-products and assessment of mutagenicity of chlorinated and brominated swimming pool water. Environ Health Perspect.

[b7-ehp-118-a466] Richardson SD, Plewa MJ, Wagner ED, Schoeny R, DeMarini DM (2007). Occurrence, genotoxicity, and carcinogenicity of regulated and emerging disinfection by-products in drinking water: a review and roadmap for research. Mutat Res.

